# A Unique TGFB1-Driven Genomic Program Links Astrocytosis, Low-Grade Inflammation and Partial Demyelination in Spinal Cord Periplaques from Progressive Multiple Sclerosis Patients

**DOI:** 10.3390/ijms18102097

**Published:** 2017-10-05

**Authors:** Serge Nataf, Marc Barritault, Laurent Pays

**Affiliations:** 1 Univ Lyon, CarMeN laboratory, Inserm U1060, INRA U1397, Université Claude Bernard Lyon 1, INSA Lyon, Charles Merieux Medical School, F-69600 Oullins, France; laurent.pays@univ-lyon1.fr; 2 Banque de Tissus et de Cellules des Hospices Civils de Lyon, Hôpital Edouard Herriot, Place d’Arsonval, F-69003 Lyon, France; 3 Univ Lyon, Department of Cancer Cell Plasticity, Cancer Research Center of Lyon, INSERMU1052, CNRS UMR5286, University Claude Bernard Lyon 1, 151 Cours Albert Thomas, 69003 Lyon, France; marc.barritault@chu-lyon.fr; 4 Service d’Anatomie Pathologique, Hospices Civils de Lyon, Groupement Hospitalier Est, 59 boulevard Pinel, 69677 Bron, France

**Keywords:** multiple sclerosis, neuroinflammation, astrocytes, myelin, bioinformatics

## Abstract

We previously reported that, in multiple sclerosis (MS) patients with a progressive form of the disease, spinal cord periplaques extend distance away from plaque borders and are characterized by the co-occurrence of partial demyelination, astrocytosis and low-grade inflammation. However, transcriptomic analyses did not allow providing a comprehensive view of molecular events in astrocytes vs. oligodendrocytes. Here, we re-assessed our transcriptomic data and performed co-expression analyses to characterize astrocyte vs. oligodendrocyte molecular signatures in periplaques. We identified an astrocytosis-related co-expression module whose central hub was the astrocyte gene *Cx43*/*GJA1* (connexin-43, also named gap junction protein α-1). Such a module comprised *GFAP* (glial fibrillary acidic protein) and a unique set of transcripts forming a *TGFB*/*SMAD1*/*SMAD2* (transforming growth factor β/SMAD family member 1/SMAD family member 2) genomic signature. Partial demyelination was characterized by a co-expression network whose central hub was the oligodendrocyte gene *NDRG1* (*N*-myc downstream regulated 1), a gene previously shown to be specifically silenced in the normal-appearing white matter (NAWM) of MS patients. Surprisingly, besides myelin genes, the NDRG1 co-expression module comprised a highly significant number of translation/elongation-related genes. To identify a putative cause of *NDRG1* downregulation in periplaques, we then sought to identify the cytokine/chemokine genes whose mRNA levels inversely correlated with those of *NDRG1*. Following this approach, we found five candidate immune-related genes whose upregulation associated with *NDRG1* downregulation: *TGFB1 (*transforming growth factor β 1), *PDGFC* (platelet derived growth factor C), *IL17D* (interleukin 17D), *IL*33 (interleukin 33), and *IL*12A (interleukin 12A). From these results, we propose that, in the spinal cord periplaques of progressive MS patients, TGFB1 may limit acute inflammation but concurrently induce astrocytosis and an alteration of the translation/elongation of myelin genes in oligodendrocytes.

## 1. Introduction

Multiple sclerosis (MS) is a neuroinflammatory disorder of the central nervous system (CNS) and the leading cause of permanent neurological disability in young adults [1]. While autoimmune mechanisms targeting the myelin sheath have been extensively demonstrated, there are still uncertainties regarding the pathophysiology of MS progressive forms [2,3,4,5]. Importantly, patients suffering from secondary or primary progressive multiple sclerosis (SPMS and PPMS, respectively) frequently exhibit slowly evolving spinal cord-related symptoms and studies based on magnetic resonance imaging (MRI) or neuropathological analyses demonstrated a high occurrence of spinal cord lesions as compared to patients with a relapsing-remitting form of the disease (RRMS) [6,7]. In this context, we recently described a particular type of tissue alteration in the spinal cord of MS patients with SPMS or PPMS [8]. We found that periplaques, i.e., areas that surround the borders of fully-demyelinated lesions, are characterized by a partial loss of myelin, a low-grade inflammatory process and an astrocytosis signed by a constant upregulation of the astrocyte markers *Cx43*/*GJA1* (connexin-43 also named gap junction protein α-1) and *AQP4* (aquaporin 4). Spinal cord periplaques extend distance away from the plaque borders and appear to evolve, at least in part, independently from plaque activity. However, when performing paired comparisons between periplaques and areas of adjacent normal-appearing white matter (NAWM), whole genome transcriptomic analyses did not allow providing a comprehensive picture of the molecular events driving astrocytosis, inflammation and partial demyelination in periplaques. Indeed, only 34 genes were found to be constantly upregulated in periplaques and 57 constantly downregulated. A potential caveat of the differential expression approach we selected concerns the varied levels of molecular alterations that may occur in areas of NAWM [9,10]. In particular, previous studies demonstrated that NAWM in MS brains or spinal cords exhibit: (i) signs of low-grade inflammation [9,10] and astrocytosis [11,12,13]; (ii) an abnormal lipid and protein composition of myelin sheaths [14,15,16]; and (iii) an epigenetic silencing of the oligodendrocyte gene *NDRG1* (*N*-myc downstream regulated 1) [17]. Thus, when performing paired comparisons between periplaques and NAWM, the genomic alterations occurring in NAWM might have masked functionally-relevant molecular events taking place in both periplaques and NAWM.

In the present paper, we re-assessed our transcriptomic data with the aim of identifying gene co-expression networks that would link astrocytosis, inflammation and myelin alterations in spinal cord periplaques. In a first step, we identified *Cx43*/*GJA1* as the only astrocyte-specific hub gene among genes that constantly co-upregulated in periplaques. To identify a molecular signature of astrocytosis in periplaques, we then used *Cx43*/*GJA1* as a “bait” and determined the top-200 genes that more closely co-upregulated with *Cx43*/*GJA1*. Interestingly, besides *GFAP* (glial fibrillary acidic protein), this *Cx43*/*GJA1* genomic module comprised the two cytokines *IL17D* (interleukin 17D) and *IL33* (interleukin 33) and a set of genes forming a TGFB/SMAD1/SMAD2 progliotic signature (transforming growth factor β/SMAD family member 1/SMAD family member 2). In parallel, we identified *NDRG1* as the only oligodendrocyte-specific hub gene among genes that constantly co-downregulated in spinal cord periplaques. To identify a molecular signature of oligodendrocytes in periplaques and NAWM, we then used *NDRG1* as a “bait” and determined the top-200 genes that more closely co-downregulated with *NDRG*1. Interestingly, *NDRG1* co-expression module comprised not only the myelin genes *PLP1* (proteolipid protein 1), *MBP* (myelin basic protein) and *MOBP* (myelin-associated oligodendrocyte basic protein), but also a highly significant number of transcripts involved in the process of mRNA translation and elongation. Finally, to unravel molecular mechanisms potentially linking partial demyelination to inflammation, we established a list of 193 candidate genes coding for cytokines, chemokines or growth factors from which we identified the top five genes whose mRNA levels inversely correlated with those of *NDRG1* in periplaques. We found that *NDRG1* mRNA levels inversely correlated with *TGFB1 (*transforming growth factor β 1)*, IL33* (interleukin 33), *IL17D* (interleukin 17D), *IL12A* (interleukin 12A) and *PDGFC* (platelet derived growth factor C).

## 2. Results

### 2.1. Workflow and Results Summary

**Figure 1:**
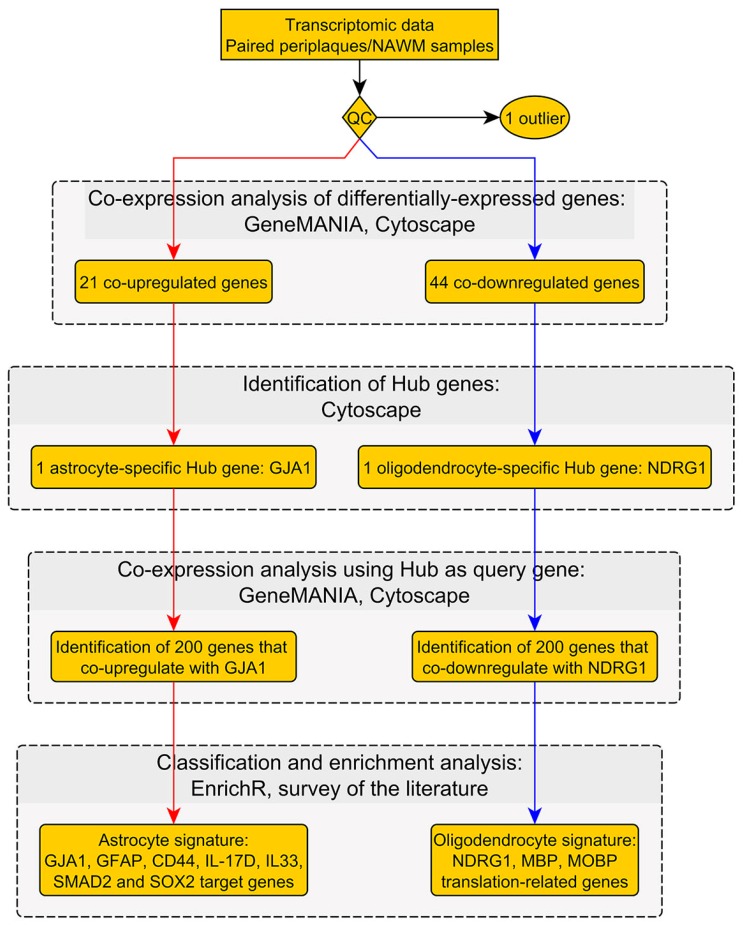
Workflow No. 1.

**Figure 2:**
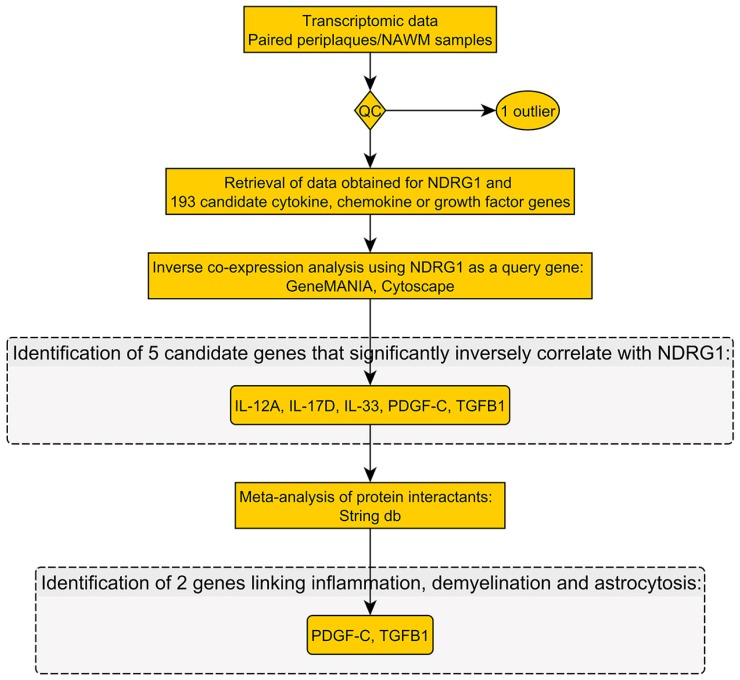
Workflow No. 2.

Our workflow and main results are summarized in Figure 1 and Figure 2.

### 2.2. Identification of Cx43/GJA1 as an Upregulated Astrocyte-Related Hub Gene in Spinal Cord Periplaques

**Figure 3:**
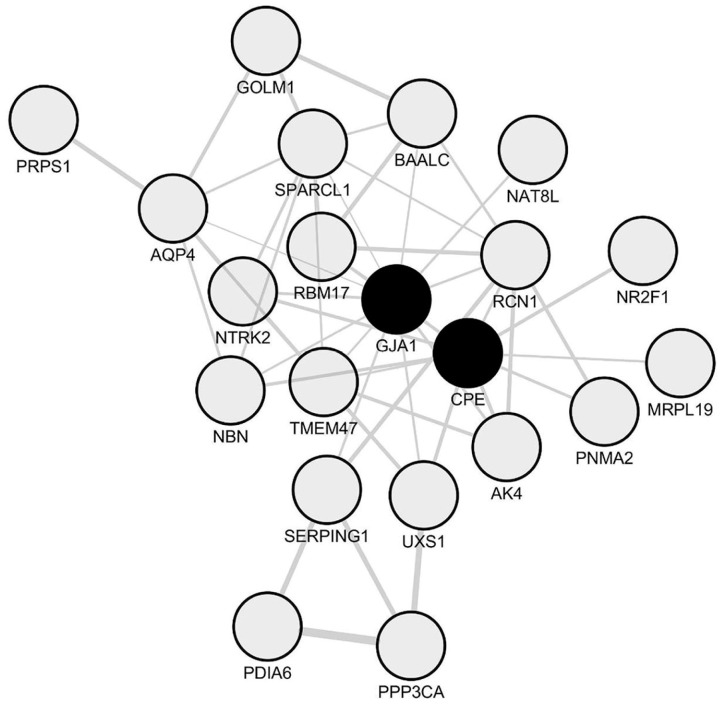
Network of co-upregulated genes in spinal cord periplaques. A co-expression network was generated with the set of genes that are constantly upregulated in periplaques as compared to adjacent normal-appearing white matter (NAWM). In this co-expression module, hubs were defined as genes (nodes) being connected to at least half of the total number of genes (nodes) forming the network. Two hub genes (black-filled nodes) were identified, namely *CPE* (Carboxypeptidase E) and the astrocyte-specific gene marker *Cx43*/*GJA1* (gap junction protein α 1 also named connexin 43). Genes are designated by their gene symbols.

Based on the data we previously obtained by paired comparisons of periplaques vs. NAWM transcriptomic profiles, we assessed whether the 34 coding genes identified as constantly upregulated in periplaques were indeed co-upregulated in periplaques. We identified a set of 21 co-upregulated mRNA species among which *Cx43*/*GJA1* (Gap junction protein α 1 also named connexin 43) was the only astrocyte-related hub gene (Figure 3). Importantly, Cx43 on astrocytes was previously reported to be upregulated in the NAWM of MS patients as compared to controls [18].

### 2.3. Identification of NDRG1 as a Downregulated Oligodendrocyte-Related Hub Gene in Spinal Cord Periplaques

**Figure 4:**
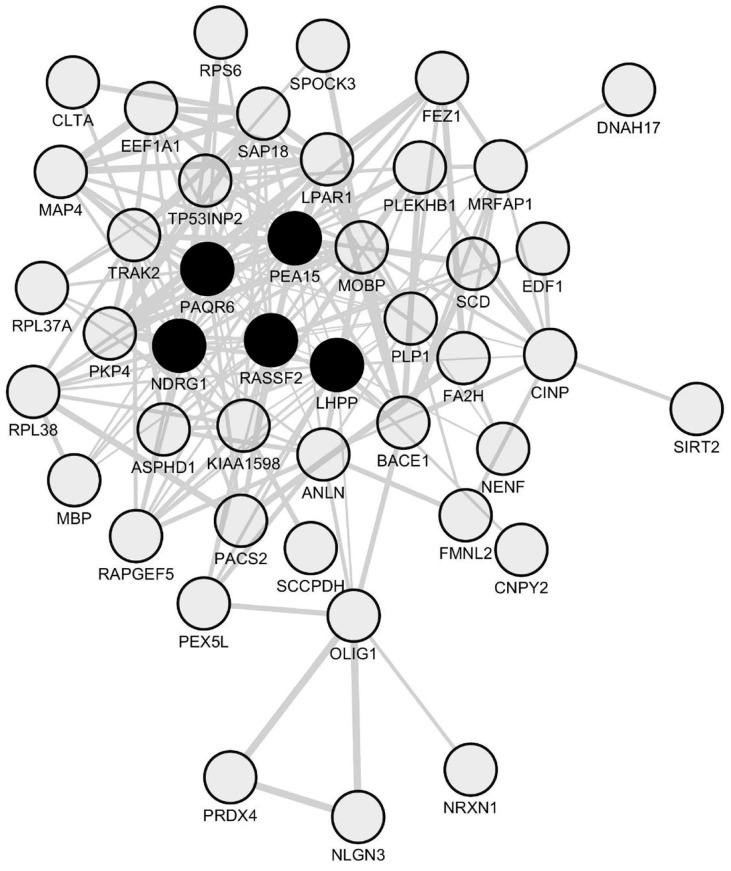
Network of co-downregulated genes in spinal cord periplaques. A co-expression network was generated with the set of genes that are constantly downregulated in periplaques as compared to adjacent normal-appearing white matter (NAWM). In this co-expression module, hubs were defined as genes (nodes) being connected to at least half of the total number of genes (nodes) forming the network. Five hub genes (black-filled nodes) were identified, namely *NDRG1* (*N*-myc downstream regulated 1), *PEA15* (phosphoprotein enriched in astrocytes 15), *RASSF2* (Ras association domain family member 2), *PAQR6* (progestin and adipoQ receptor family member 6) and *LHPP* (phospholysine phosphohistidine inorganic pyrophosphate phosphatase). Genes are designated by their gene symbols. Full names of functionally-relevant genes are provided in Table 1.

We then assessed whether the 57 coding genes identified as constantly downregulated in paired comparisons of periplaques vs. NAWM were co-downregulated in periplaques. We identified a set of 44 co-downregulated genes (Figure 4 and Table 1) among which NDRG1 was the only hub gene previously recognized as being specifically expressed by oligodendrocytes in the central nervous system [17,19,20].

### 2.4. A Unique Set of Genes Co-Upregulate with CX43/GJA1 in Periplaques of MS Spinal Cords

**Figure 5:**
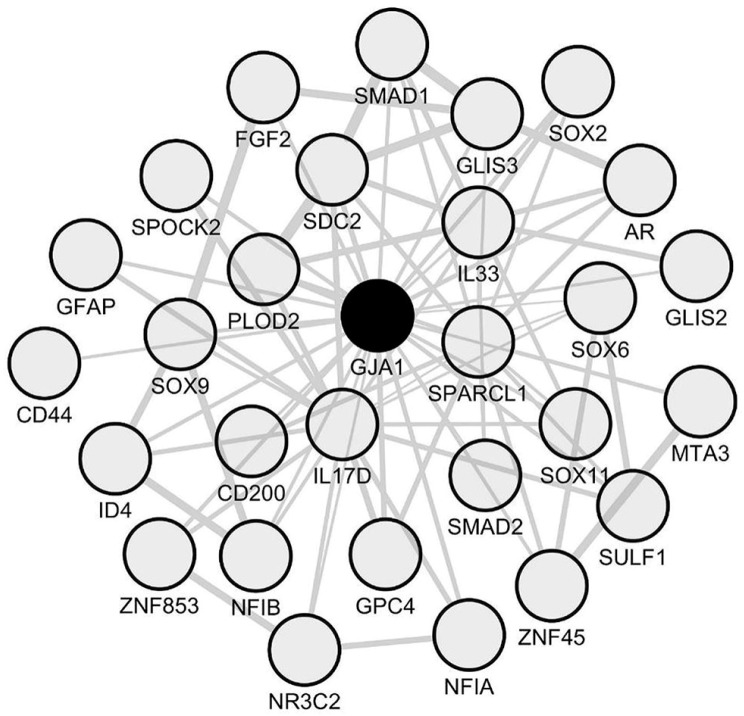
Astrocyte signature inferred from the *GJA1*/*Cx43* co-expression module in spinal cord periplaques. To identify an astrocyte molecular signature in spinal cord periplaques, we used the astrocyte-specific hub gene *Cx43*/*GJA1* as a “bait” and retrieved the top-200 mRNA species that most closely co-upregulated with *Cx43*/*GJA1*. The co-expression network formed by functionally-relevant genes is shown. Genes are designated by their gene symbols. Gene full names are provided in Table 2.

To identify a putative astrocyte molecular signature in periplaque areas of MS spinal cords, we used the astrocyte-specific hub gene *Cx43*/*GJA1* as a “bait” and retrieved the top-200 mRNA species that more closely co-expressed with *Cx43*/*GJA1* in our whole set of data. Functionally-relevant genes are shown in Table 2 and Figure 5. The full list of *Cx43*/*GJA1* co-expressed genes is provided in Supplementary Data S1.

Besides *GFAP*, such a genomic network comprised three groups of genes that we found of particular relevance in the context of astrocytosis, inflammation and tissue remodeling: (i) transcription factors (TFs); (ii) cytokines; and (iii) genes coding for extra-cellular matrix (ECM) molecules (Figure 3 and Table 2). Interestingly, the TFs that co-expressed with *Cx43*/*GJA1* comprised *SMAD1* and *SMAD2*, which are two major signaling components of the TGF-β and Bone Morphogenetic Proteins (BMPs) pro-gliotic pathways [21,22,23,24]. Moreover, TFs that co-expressed with *Cx43*/*GJA1* also comprised *SOX2* a stem cell-related gene expressed by proliferating astrocytes [25,26] and NFAI, a positive regulator of GFAP transcription [27]. On the other hand, the androgen receptor (*AR*), which was also found to co-express with *Cx43*/*GJA1*, was previously shown to inhibit reactive astrocytosis under varied CNS conditions [28,29,30,31,32,33]. Of note, *AR* co-expressed also with *Cx43*/*GJA1* when excluding the two male patients from our analysis (Supplementary Data S2). To establish a potential link between *Cx43*/*GJA1* co-expressed TFs and other genes of the *CX43*/*GJA1* module, we performed an enrichment analysis using ChEA2016, a Chip-Seq and Chip-Chip database and webtool [34]. We found that the *GJA1*/*Cx43* module was significantly enriched (adjusted *p*-value < 0.01) in previously identified gene targets of AR or SOX2 in human cells (Supplementary Data S3). However, the *GJA1*/*Cx43* module was not enriched in known functional pathways as assessed with the Reactome 2016 database and webtool [35]. Surprisingly, we found that only two cytokines, namely IL17D and IL33, co-upregulated with *Cx43*/*GJA1* in spinal cord periplaque areas. Other inflammation-related genes comprised notably *CD44* and *CD200* that were both previously demonstrated on reactive astrocytes [36,37]. Finally, the *GJA1*/*Cx43* module comprised several ECM-related genes that are expressed by reactive astrocytes: *GPC4* [38], *SULF1* [39] and *SPARCL1* [40].

### 2.5. A Unique Set of Genes Co-Express with NDRG1 in Periplaque Areas of MS Spinal Cords

**Figure 6:**
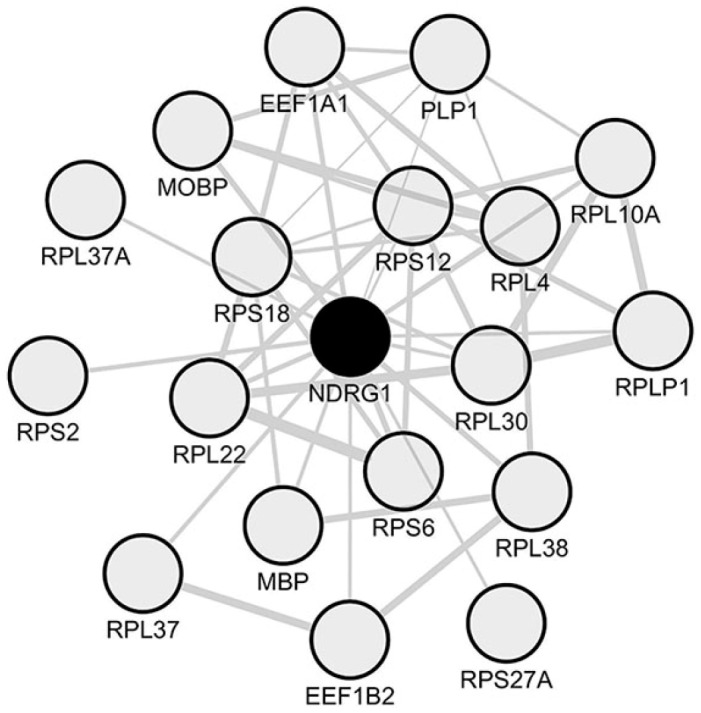
Oligodendrocyte signature inferred from the *NDRG1* co-expression module in spinal cord periplaques. To identify an oligodendrocyte molecular signature in spinal cord periplaques, we used the oligodendrocyte-specific hub gene *NDRG1* (*N*-myc downstream regulated 1) as a “bait” and retrieved the top-200 mRNA species that most closely co-downregulated with *NDRG1*. The co-expression network formed by functionally-relevant genes is shown. Genes are designated by their gene symbols. Gene full names are provided in Table 3.

To unravel a molecular signature of oligodendrocytes in periplaque areas of MS spinal cords, we used the oligodendrocyte-related hub gene *NDRG1* as a “bait” and retrieved the top-200 mRNA species that more closely co-expressed with *NDRG1*. The full list of *NDRG1* co-expressed genes is provided in Supplementary Data S4. As expected, such a module comprised genes coding for myelin proteins, namely *MBP*, *MOBP* and *PLP1*. Similarly to the *Cx43*/*GJA1* module, the *NDRG1* module also comprised several TFs. However, the network of *NDRG1* co-expressed genes was not enriched in known targets of these TFs, as assessed with the ChEA2016 database and webtool. A survey of the “TargetScan microRNA” [41] database showed that the *NDRG1* co-expression module was not significantly enriched in known targets of miRNAs. However, pathway analysis with the webtool “Reactome 2016” showed a highly significant enrichment in genes involved in the functional pathway “Eukaryotic Translation Elongation” (Reactome 2016, adjusted *p*-value = 37 × 10^−9^) (Figure 6 and Table 3).

This finding appears of particular interest since, in oligodendrocytes, the translation of *MBP* and *MOBP* was previously shown to rely on specific mechanisms requiring a transport of the protein translation machinery (including ribosomal proteins) along the cell processes of oligodendrocytes [42,43,44,45,46,47]. To explore the existence of potential links between *NDRG1* and the processes of translation/elongation, we performed a meta-analysis of NDRG1 protein interactants. While the STRING database allowed to recover only 20 direct protein interactants with no significant enrichment relating with translation/elongation, the Wiki-Pi database, gathering data from high throughput technologies, listed 64 NDRG1 interactants (Supplementary Data S5) that showed a highly significant enrichment in the “Translation” pathway (Reactome 2016, adjusted *p*-value = 4.80 × 10^−11^). Similar results were obtained when assessing NDRG1 interactants in the protein interactome database TissueNet v.2 [48] (Supplementary Data S5). This result points to a potential link between NDRG1 downregulation in oligodendrocytes and a subsequent altered translation/elongation of myelin genes leading to partial demyelination.

### 2.6. Identification of Candidate Soluble Factors that May Trigger NDRG1 Silencing in Periplaque Areas of MS Spinal Cords

**Figure 7:**
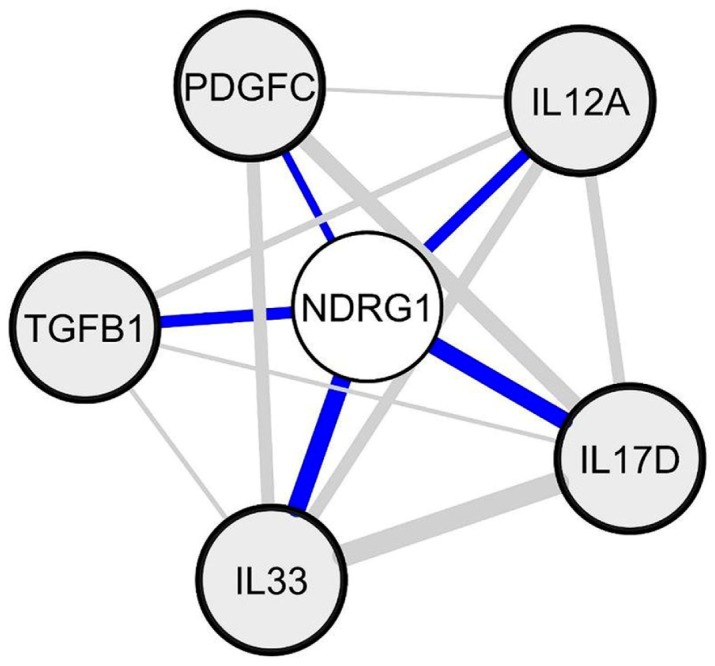
Identification of candidate soluble factors that putatively repress *NDRG1* expression. Starting from a list of 193 candidate genes coding for cytokines, chemokines or growth factors, we used the GeneMANIA software to identify mRNA species whose levels inversely correlated with those of *NDRG1* (*N*-myc downstream regulated 1) in spinal cord periplaques. The top five genes whose expression levels inversely correlated with *NDRG1* were: *TGFB1* (transforming growth factor β 1)*, IL33* (interleukin 33), *IL17D* (interleukin 17D), *IL12A* (interleukin 12A) and *PDGFC* (platelet derived growth factor C).

While a previous study showed that *NDRG1* is specifically silenced in the NAWM of MS patients [17], we found that *NDRG1* was constantly downregulated in spinal cord periplaques as compared to adjacent NAWM. We thus sought to identify candidate soluble factors (in particular immune molecules) that would be potentially involved in a repression of *NDRG1* expression in periplaque areas of MS spinal cords. To this aim, we first established a list of 193 candidate genes coding for cytokines, chemokines or growth factors. We then used the GeneMANIA software to identify the top five candidate genes whose mRNA levels harbored the more closely inverse correlation with *NDRG1* mRNA levels in our whole set of data. These top five genes were established as follows: *IL17D*, *IL33*, *IL12A*, *PDGF-C* and *TGFB1* (Figure 7).

### 2.7. Assessment in Normal Human Astrocytes of Genes Associated with the Identified Cx43/GJA1 Module

We sought to determine whether the genes forming the functional core of the *Cx43*/*GJA1* co-expression module were also expressed in normal human astrocytes. To this aim, we assessed the human CNS RNA-Seq database published by the Ben Barres group [49,50] in which mRNA levels were obtained by RNA-Seq from highly-purified CNS cell types (astrocytes, neurons, endothelial cells, and oligodendrocytes) extracted from normal human brains. Using this database, we found that, out of the 28 genes forming the functional core of the *CX43*/*GJA1* module (depicted in Figure 5 and Table 2), 24 were significantly enriched in mature human astrocytes as compared to other mature CNS cells (Supplementary Data S6); three genes for which values were not provided could not be assessed and only one gene (SULF1) was not statistically enriched in human mature astrocytes. Then, starting from the 24 genes identified as enriched in mature human astrocytes, we set a threshold of 10× (in astrocytes as compared to other CNS cell types) to identify those which could be considered as astrocyte-specific. The list of such astrocyte-specific genes is presented in Table 4.

Since, based on this approach, IL17D and IL33 could be considered as astrocyte-specific in normal human mature astrocytes, we then aimed at determining whether other interleukins could be identified as being astrocyte-specific as well. Interestingly, when assessing the Ben Barres database and using again the threshold of X10 (in astrocytes as compared to other CNS cell types), we found that, out of 33 interleukins tested, only IL17D and IL33 could be considered as astrocyte-specific (Table 5 and Supplementary Data S7). In addition, among the 33 interleukins tested, we found that: (i) the highest mRNA levels as expressed in FKPM (fragments per kilobase of exon per million fragments mapped) were observed for IL17D and ILL33; and (ii) only seven interleukins could be considered as constitutively expressed when using the threshold of 0.5 FKPM (the threshold used in the Ben Barres database to determine whether or not a gene can be considered as being expressed). Finally, it is worth noting that studies from the Ben Barres group showed that the astrocyte specificity of IL17D is not observed in mice [51] meaning that functions exerted by IL17D in the human brain may not be extrapolated from studies performed in mice.

### 2.8. Assessment in Normal Human Oligodendrocytes of Genes Associated with the Identified NDRG1 Module

We sought to determine whether the genes forming the functional core of the NDRG1 co-expression module we identified were also expressed in human normal oligodendrocytes. Using the human CNS RNA-Seq database in Section 2.6, we found that, out of the 18 genes forming this functional core, 16 were statistically enriched in oligodendrocytes as compared to other CNS cells (Supplementary Data S8). Interestingly, several translation/elongation-related genes from this core module were enriched by a factor of 2–3 in oligodendrocytes as compared to other CNS cells: *RPS12* (Ribosomal protein S12), *RPL37A* (Ribosomal protein L37a), *RPS18* (Ribosomal protein S18), *RPL30* (Ribosomal protein L30), *RPL22* (Ribosomal protein L22), *RPS6* (Ribosomal protein S6), *RPL38* (Ribosomal protein L38), *RPL37* (Ribosomal protein L38) and *EEF1B2* (Eukaryotic translation elongation factor 1 β 2) (Supplementary Data S8).

### 2.9. Identification of a Molecular Pathway Linking MYCN ((MYCN proto-oncogene, bHLH transcription factor) to PDGFC and TGFB1

**Figure 8:**
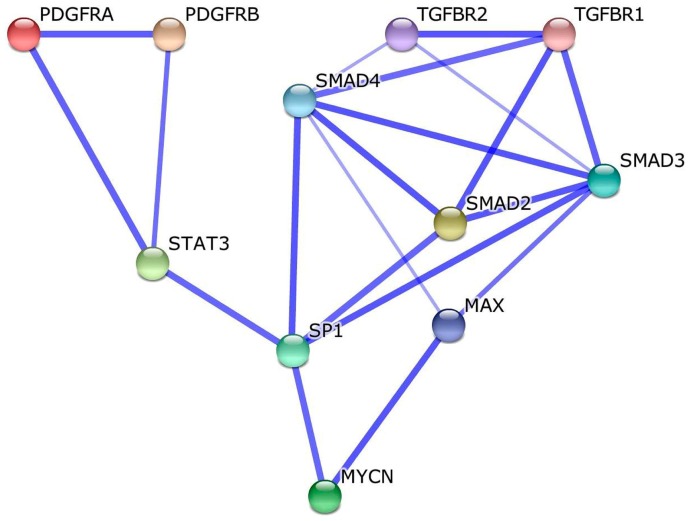
Identification of molecular pathways linking PDGFC (platelet derived growth factor C) and TGFB1 (transforming growth factor β 1) to MYCN (MYCN proto-oncogene, bHLH transcription factor). A meta-analysis of the human interactome was performed to determine whether signaling pathways could link MYCN (a known repressor of NDRG1 expression) to the identified candidate IL17D, IL33, IL12A, PDGFC and TGFB1. Data show that PDGFRA (platelet derived growth factor receptor α, a PDGFC receptor) and TGFBR1 (transforming growth factor β receptor 1, a TGFB1 receptor) are second shell interactants of MAX (Myc associated factor X) and SP1 (Sp1 transcription factor), two transcription factors that are recruited by MYCN during the transcriptional repression of specific target genes.

*NDRG1* was originally identified as a major target gene repressed by the transcription factor MYCN (also named *N*-Myc) in neuroblastoma cells [52,53]. Since then, in multiple cell types, MYCN was shown to silence *NDRG1* gene expression via a process involving a hypermethylation of the *NDRG1* promoter [52,53,54,55,56,57]. On this basis, we performed a meta-analysis of the human interactome to determine whether signaling pathways could link MYCN to the identified candidate molecules (i.e., IL17D, IL33, IL12A, PDGF-C and TGFB1). We found that PDFGRA (platelet derived growth factor receptor α, a PDGFC receptor) and TGFBR1 (transforming growth factor β receptor 1, a TGFB1 receptor) are second shell interactants of MAX (Myc associated factor X) and SP1 (Sp1 transcription factor) (Figure 8), two TFs that are recruited by MYCN during the transcriptional repression of specific target genes [54]. No direct or secondary interactions could be retrieved between MYCN and the receptors for IL33 or IL12A (the IL17D receptor being not yet identified could not be investigated).

## 3. Discussion

In this paper, gene co-expression analyses were performed in order to infer astrocytes vs. oligodendrocytes molecular signatures in spinal cord periplaques of MS patients. Regarding astrocytes, genes that were found to co-upregulate with *GJA1*/*Cx43* in periplaques formed a pro-gliotic signature which composition is compatible with a combined TGFB/SAMD1/SMAD2- and SOX2-driven program. While TGFB and SOX2 were both previously identified as astrocytosis-promoting factors [21,22,23,24], TGFB was further shown to induce an astrocyte-specific developmental program that, in turn, prevents effective remyelination in MS plaques and periplaques [58]. Superimposing the TGFB/SMAD1/SMAD2 and SOX2 progliotic signatures, we also found that the *GJA1*/*Cx43* module was characterized by a highly significant enrichment in genes that are regulated by the AR. Interestingly, even when excluding males from our study, the *AR* was still co-expressed with *GJA1*/*Cx43*. As androgens are known to prevent astrocytosis [28,29,30,31,32,33], our results raise the possibility that in males and females suffering from progressive forms of MS, activation of the AR is somehow defective and fails to downregulate a large set of progliotic genes induced by the TGFB/SMAD1/SMAD2 pathway. This could be due to an age-related decrease of circulating testosterone or of the circulating androgen precursors (Dehydroepiandrosterone and Androstendiol) that can be intracellularly metabolized into testosterone [59,60,61,62]. Another explanation, not exclusive from the former one, could be that the AR signaling pathway might be hampered or disrupted in periplaque astrocytes despite a normal level of circulating or intracrine testosterone. Supporting this view, mechanisms of mutual exclusion between AR and TGFB signaling pathways were previously demonstrated in different cell types [63,64,65,66,67,68].

With regard to inflammation, we found that, in spinal cord periplaques, the astrocytois signature formed by *GJA1*/*Cx43* gene module comprised only two cytokines: *IL17D* and *IL33*. These two cytokines were previously shown to be not only highly expressed in normal (quiescent) human mature astrocytes but also to be astrocyte-specific at least to some extent [49,50]. Deciphering the specific roles exerted by such cytokines in MS pathophysiology would deserve specific experimental approaches that would possibly need the use of human cells. Indeed, while IL17D is a pro-inflammatory cytokine directing the intra-tissular recruitment of NK T-cells in mice [69,70] (via a yet unknown receptor), its abundant expression in quiescent astrocytes appears to be specific to the human species [51]. It is worth noting that IL17D was proposed to share with other members of the IL17 family, a specific ability to induce TGFB secretion in a large range of cell types [71,72]. Interestingly also, the astrocytic expression of IL33 was recently demonstrated in MS plaques and IL33 was shown to inhibit the de novo myelination of rat axons in vitro [73]. Nevertheless, other works reported that IL33 promotes remyelination [74] and exerts potent anti-inflammatory and neuroprotective functions [75]. Again, discrepancies between results could possibly be explained by species-specific differences, notably regarding the expression profile of IL1RL1 (also named ST2), the receptor to IL33 [76,77]. In particular, when comparing the constitutive expression of IL1RL1 in murine vs. human CNS cells, IL1RL1 is exclusively expressed by endothelial cells in human cells while its expression is limited to macrophages/microglia in murine cells. Along this line, the pleiotropic effects of IL33 on immune cells were shown to be regulated by a finely-tuned and cell-specific expression of IL1RL1, notably on T-cells [76,77]. Finally, besides TFs and immune genes, the *GJA1*/*Cx43* module included several key extra-cellular matrix (ECM)-related genes involved in tissue remodeling. Among these, Procollagen Lysyl Hydroxylase 2 (*PLOD2*) is a TGFB-induced enzyme [78,79,80] that promotes fibrosis via the crosslinking of collagen molecules [81,82,83].

The second aim of our work was to identify a molecular signature of oligodendrocytes in the spinal cord periplaques of MS patients. Among the network of genes that were constantly downregulated in periplaques, *NDRG1* was identified as the only oligodendrocyte-specific hub gene. Such a finding has to be interpreted in light of a recent work demonstrating that *NDRG1* is also the only oligodendrocyte-specific gene whose silencing was demonstrated in the NAWM of MS patients. These data, while further supporting the role of *NDRG1* as a master regulator of oligodendrocyte differentiation and myelin maintenance [17,19,20], suggest also that *NDRG1* may be a major target of a yet uncharacterized process leading to diffuse myelin alterations in periplaques. When using *NDRG1* as a bait gene to identify a large co-expression module in oligodendrocytes, we found that only three genes coding for myelin proteins co-downregulated with NDRG1 in periplaques: *MBP*, *MOBP* and *PLP1.* Interestingly, as opposed to the GJA1/Cx43 module, no specific enrichment in known targets of co-expressed TF could be evidenced in the *NDRG1* module. Moreover, we were neither able to identify enrichment in apoptosis-related pathways nor in miRNA targets, which would have provided molecular schemes explaining oligodendrocyte cell loss or altered myelin gene expression. In contrast, the *NDRG1* co-expression module was highly significantly enriched in genes involved in translation/elongation of mRNAs. Although, at this stage, one may only extrapolate on the significance of such an enrichment, it is important to underscore that the translation of both *MBP* and *MOBP* mRNAs in oligodendrocytes rely on a unique mechanism during which various components of the protein translation machinery (including ribosomal proteins) are transported along the cell processes of oligodendrocytes [42,43,44,45,46,47]. Strikingly, when performing a meta-analysis of human NDRG1 protein interactants, we found a highly significant enrichment in proteins involved in the “Translation” pathway (Reactome 2016, adjusted *p*-value = 1.80 × 10^−11^) (Supplementary Data S5). On this basis, one may thus propose that *NDRG1* silencing in oligodendrocytes may hamper myelination via the coordinated downregulation of a specific subset of ribosomal genes supporting the translation/elongation of *MBP* and *MOBP* mRNAs.

Finally, we attempted to identify candidate soluble molecules that could be responsible for *NDRG1* downregulation in spinal cord periplaques. Our analysis pointed to a network of five genes whose expression levels inversely correlated with those of *NDRG1* with the highest levels of confidence: (i) *IL17D* and *IL33*, the two cytokines found to co-upregulate with GJA1/Cx43 in periplaques; (ii) *IL12-A*, a potent inducer of TH1 (T-Helper 1) polarization [84]; (iii) *PDGFC* (Platelet-derived growth factor C), a profibrotic gene [85,86] and a regulator of the neurovascular unit [87]; and (iv) *TGFB1* (Transforming growth factor-1). In fact, these correlations do not allow establishing a functional causative link between an upregulation of these cytokines and the downregulation of NDRG1 expression. Again, we used bioinformatics tools to explore such putative links and performed a meta-analysis of the protein interactants potentially linking the identified candidate cytokines with *N*-Myc. Indeed, a well identified mechanism of *NDRG1* repression relies on the binding of *N*-Myc to the promoter region of *NDRG1* and the subsequent recruitment of histone deacetylases leading to promoter hypermethylation and an epigenetic silencing of NDRG1 [52,53,54,55,56,57]. Our meta-analysis showed that only TGFBR1 (an oligodendrocyte-expressed receptor to TGFB1) and PDGRA (an oligodendrocyte-expressed receptor to PDGFC) formed a close (second shell) protein network with *N*-MyC. Overall, since the *NDRG1* promoter was shown to be hypermethylated in the NAWM of MS patients [17], our results indicate that TGFB1 and/or PDGFC released in MS periplaques could be responsible for a silencing of *NDRG1* in periplaques and, to a lesser extent, in the NAWM.

Overall, our work points to a major role of TGFB1 in the development of astrocytosis and diffuse myelin alterations in the spinal cord periplaques of MS patients. In particular, we propose a new pathophysiological scheme where TGF-β1 would be central to a self-perpetuated process during which: (i) IL17-D, synthesized by reactive astrocytes, triggers the production of TGFB1; and (ii) astrocyte-derived TGFB1 fuels chronic gliosis and hamper myelination via a transcriptional repression of *NDRG1*. Thus, blocking TGFB1 could possibly reverse the TGFB1-induced downregulation of *NDRG1* in oligodendrocytes, allowing a physiological level of translation/elongation of myelin genes to be achieved in oligodendrocytes. Supporting the hypothesis of a reversible dysfunction of oligodendrocyte in progressive MS, recent studies showed that biotin, an activator of myelin synthesis, exerted therapeutic effects on progressive MS patients when administered at high doses [88,89]. While further studies are needed to test the role of TGFB1 in MS pathophysiology, it is important to notice that the monoclonal antibody Fresolimumab directed against all TGFB isoforms is available for clinical trials [90], cross the blood-brain barrier [91] and is currently under evaluation in patients suffering from systemic sclerosis [92]. Finally, one may keep in mind that, although TGFB1 is generally considered as an overall anti-inflammatory cytokine, robust in vivo data previously showed that overexpression of TGFB1 in the CNS aggravates experimental autoimmune encephalitis [93,94].

## 4. Materials and Methods

### 4.1. Transcriptomic Data

Co-expression networks were generated from a previously published set of transcriptomic data that our group obtained by whole genome analyses of paired NAWM and periplaque samples derived from 8 SPMS patients [8]. Data are available at the public gene array repository bank GEO DataSets (Ref: GSE52139). To ensure the highest level of confidence to co-expression analyses, we first submitted transcriptomic data to a stringent quality control using the arrayQualityMetrics Bioconductor package (v3.28.2) [95] in R (v3.3.0). We removed from further analyses the transcriptomic profile obtained from 1 out of 16 samples as it was detected as an outlier by both boxplots and MA plots criteria.

### 4.2. Generation of a Global Co-Expression Network

An expression file was generated that comprised an “OBJECT” column in which gene symbols were provided for each row and 15 “SAMPLE” columns compiling normalized data values for each sample and each transcript. This expression file was then imported in the GeneMANIA Cytoscape plugin (version 3.3.4 data version 2014-08-02-core) [96,97] allowing the generation of a co-expression network that, based on the Pearson correlation test, gathered 735,240 interactions.

### 4.3. Connectivity Analysis of Gene Co-Expression Networks

Starting from the global co-expression network generated from periplaque and NAWM samples, we investigated the correlation links that could be established between genes that were previously found to be differentially regulated in paired comparisons between periplaques and NAWM samples. Using the GeneMANIA plugin in Cytoscape software, 2 sets of genes were entered separately as independent queries: (i) the 34 genes found to be constantly upregulated in periplaques [8]; and (ii) the 57 genes found to be constantly downregulated in periplaques [8]. For each analysis, the number of additional connected genes to be identified was set to zero in order to limit the search for co-expression links to query genes only. Then, to identify hub genes among these modules, we used the “Network analysis” tool in Cytoscape software. A hub gene was defined as a gene (node) being connected to at least half of the total number of genes (nodes) forming the network.

### 4.4. Identification and Analysis of the Cx43/GJA1 Co-Upregulated Gene Network

Starting from the global expression network loaded on Cytoscape/GeneMANIA, *CX43*/*GJA1* (*GJA1*) was entered as a query gene, and the number of connected genes to be identified was set to 200. The generated list was then assessed with regard to the presence of genes considered as functionally relevant in the context of astrocytosis, inflammation and tissue remodeling. These notably included genes coding for: (i) cytokines; (ii) extracellular matrix (ECM); or (iii) transcription factors (TF). Identification of functionally-relevant genes was performed by a survey of the literature, on the basis of GO terms annotation, and using the TF database “AnimalTFDB” [98]. Finally, the 200 genes forming the *Cx43/GJA1* co-expression network were also analyzed with regard to their enrichment in: (i) genes involved in specific pathways; (ii) genes identified as known targets of human transcription factors (TF); and (iii) genes identified as known targets of miRNA. These enrichment analyses were performed using the EnrichR webtools [99]: “ChEA2016” [34] (allowing to survey a total of 622 ChIP-ChIP or ChIP-seq sets of data that were manually curated), “TargetScan microRNA” [41] and “Reactome 2016” [35].

### 4.5. Identification and Analysis of the NDRG1 Co-Downregulated Gene Network

Starting from the global expression network loaded on Cytoscape/GeneMANIA, *NDRG1* was entered as a query gene, and the number of connected genes to be identified was set to 200. The generated list was then assessed with regard to the presence of genes coding for myelin or oligodendrocyte-related molecules as determined by GO terms annotation and a survey of the literature. The complete list of genes forming the *NDRG1* co-expression network was also analyzed with regard to its enrichment in: (i) genes involved in specific pathways; (ii) genes identified as known targets of human transcription factors (TF); and (iii) genes identified as known targets of miRNA. These enrichment analyses were performed using the open source website EnrichR [99] and the same webtools as described above.

### 4.6. Identification of aNetwork of NDRG1 Inversely Correlated Genes Coding for Cytokines/Chemokines or Growth Factors

As GeneMANIA recognizes only positive correlation links, its use for the identification of mRNA species that inversely correlate with a given query gene requires the prior replacement of the corresponding mRNA values by opposite values. To identify a network of cytokines, chemokines or growth factors that inversely correlated with *NDRG1*, we first established a list of 193 candidate genes (Supplementary Data S9) that included notably 38 interleukins, 17 interferons, 36 chemokines, 35 growth factors, 22 metalloproteases, 6 angiogenic factors and 5 granzymes. GeneMANIA was then loaded with an expression file that comprised: (i) the original values obtained for each of the probes corresponding to the above-described list of cytokines/chemokines or growth factors; and (ii) the values opposite to the original ones obtained with the NDRG1 probe. Again, the number of additional connected genes to be identified was set to zero. *NDRG1* was entered as a query gene, and the number of connected genes to be identified was set to 10. As GeneMANIA was conceived to process large sets of data, its algorithms include a sparsification process during which only the 50 strongest correlation links for each object are retained [96]. Therefore, for expression files comprising a low number of objects, correlation links being ranked in the top 50 might still not reach significance. To circumvent this possible drawback, for each of the 10 genes that were initially identified as inversely correlated with *NDRG1* with the GeneMANIA software, the statistical significance of the inverse correlation was checked using the Pearson correlation test. Only genes for which *p* values were ≤0.01 were taken into account.

### 4.7. Meta-Analysis of MYCN Protein Interactants

When needed, the open source websites “STRING” [100] and Wiki-Pi [101] were used to perform meta-analyses of protein-protein interactions. STRING allows protein interaction networks to be generated via a survey of distinct databases that list known protein interactions from published and manually-curated biochemical studies [100]. Here, we used STRING to identify interactants that would link MYCN to the receptors IL12RB1, IL12RB2, IL1RL1 (IL33 receptor), PDGFRA, PDGFRB (platelet derived growth factor receptor β, a PDGFC receptor), TGFBR1, TGFBR2 (transforming growth factor β receptor 2, a TGFB1 receptor) and TGFBR3 (transforming growth factor β receptor 3, a TGFB1 receptor). Several round of analyses were performed in which two query proteins were entered for each analysis: MYCN and one of the above-mentioned receptors. The number of first-shell protein interactants to be identified was set to 500 and only high confidence interactions (score ≥ 0.7 according to the classification provided by the STRING website) retrieved from published and manually-curated biochemical studies were taken into account. Selected interaction networks were then loaded in Cytoscape in order to facilitate the visualization of identified pathways. In another set of analyses, we used the Wiki-Pi database to retrieve NDRG1 protein interactants. Wiki-Pi is a resource that is not manually-curated but allows a large survey of both Biogrid [102] and HPRD [103] databases obtained by high throughput technologies. Wiki-Pi compiles 48419 unique binary biophysical interactions among 10,492 human proteins.

## Figures and Tables

**Table 1:** Hub genes and oligodendrocyte-related genes that co-downregulate in spinal cord periplaques as compared to NAWM.

**Hub Genes**	
*LHPP*	Phospholysine phosphohistidine inorganic pyrophosphate phosphatase
*NDRG1*	*N*-myc downstream regulated 1
*PAQR6*	Progestin and adipoQ receptor family member VI
*PEA15*	Phosphoprotein enriched in astrocytes 15
*RASSF2*	Ras association (RalGDS/AF-6) domain family member 2
**Oligodendrocyte-Related Genes**	
*NDRG1*	*N*-myc downstream regulated 1
*MOBP*	Myelin-associated oligodendrocyte basic protein
*MBP*	Myelin basic protein
*PLP1*	Proteolipid protein 1
*OLIG1*	Oligodendrocyte transcription factor 1
*SIRT2*	Sirtuin (silent mating type information regulation 2 homolog) 2 (*Saccharomyces cerevisiae*)
*FA2H*	Fatty acid 2-hydroxylase

A co-expression network was generated with the set of genes that constantly downregulated in periplaques as compared to adjacent NAWM. In this co-expression module, hubs were defined as genes (nodes) being connected to at least half of the total number of genes (nodes) forming the network.

**Table 2:** List of functionally-relevant genes that co-express with *GJA1*/*Cx43* in spinal cord periplaque areas.

	**Transcription Factors**
*AR*	Androgen receptor
*NR3C2*	Nuclear receptor subfamily 3 group C member 2 (mineralocorticoid receptor)
*SMAD1*	SMAD family member 1
*SMAD2*	SMAD family member 2
*SOX2*	SRY (sex determining region Y)-box 2
*SOX6*	SRY (sex determining region Y)-box 6
*SOX9*	SRY (sex determining region Y)-box 9
*SOX11*	SRY (sex determining region Y)-box 11
*ZNF45*	Zinc finger protein 175
*ZNF853*	Zinc finger protein 853
*GLIS2*	GLIS family zinc finger 2
*GLIS3*	GLIS family zinc finger 3
*NFIA*	Nuclear factor I/A
*NFIB*	Nuclear factor I/B
*ID4*	Inhibitor of DNA binding 4, dominant negative helix-loop-helix protein
*MTA3*	Metastasis associated 1 family, member 3
	**Cytokines**
*IL33*	Interleukin 33
*IL17D*	Interleukin 17D
	**ECM-Related Genes**
*SPARCL1*	SPARC-like 1 (hevin)
*SDC2*	Syndecan 2
*PLOD2*	Procollagen-lysine, 2-oxoglutarate 5-dioxygenase 2
*GPC4*	Glypican 4
*SULF1*	Sulfatase 1
*SPOCK2*	SPARC/osteonectin, cwcv and kazal-like domains proteoglycan (testican) 2
	**Others**
*GFAP*	Glial fibrillary acidic protein
*CD44*	CD44 molecule (Indian blood group)
*CD200*	CD200 molecule
FGF2	Fibroblast growth factor 2 (basic)

A co-expression analysis was performed in which the astrocyte-specific gene CX43/GJA1 was used as a “bait” to identify a putative molecular signature of reactive astrocytes in periplaque areas of MS spinal cords. The top-200 genes that more closely co-expressed with *CX43/GJA1* were identified. Listed are genes considered as functionally-relevant in the context of astrocytosis and inflammation.

**Table 3:** List of functionally-relevant genes that co-express with *NDRG1* in spinal cord periplaque areas.

	**Myelin-Related Genes**
*MBP*	Myelin basic protein
*MOBP*	Myelin-associated oligodendrocyte basic protein
*PLP1*	Proteolipid protein 1
	**Genes Involved in the “Eukaryotic Translation Elongation” Pathway (Reactome 2016, *p* = 1.37 × 10^−9^)**
*EEF1A1*	Eukaryotic translation elongation factor 1 α 1
*EEF1B2*	Eukaryotic translation elongation factor 1 β 2
*RPLP1*	Ribosomal protein, large, P1
*RPL4*	Ribosomal protein L4
*RPL10A*	Ribosomal protein L10a
*RPL22*	Ribosomal protein L22
*RPL30*	Ribosomal protein L30
*RPL37*	Ribosomal protein L37
*RPL37A*	Ribosomal protein L37a
*RPL38*	Ribosomal protein L38
*RPS2*	Ribosomal protein S2
*RPS6*	Ribosomal protein S6
*RPS12*	Ribosomal protein S12
*RPS18*	Ribosomal protein S18
*RPS27A*	Ribosomal protein S27A

A co-expression analysis was performed in which the oligodendrocyte-specific gene *NDRG1* (*N*-myc downstream regulated 1) was used as a “bait” to identify a putative molecular signature of oligodendrocytes in spinal cord periplaques. The top-200 genes that more closely co-express with *NDRG1* in spinal cord periplaque areas were identified. Listed are genes considered as functionally-relevant in the context of myelin loss.

**Table 4:** List of functionally-relevant genes that co-express with *Cx43*/*GLA1* in spinal cord periplaque areas and harbor an astrocyte-specific profile in normal mature human astrocytes.

Gene Symbol	Astrocytes (Mean FPKM)	SD	Other CNS Cells (Mean FPKM)	SD	Fold Change	*p*-Value
*FGF2*	15.9	4.1	0.9	0.6	16.9	5.40 × 10^−5^
*GLIS3*	5.5	1.7	0.3	0.3	16.1	5.29 × 10^−5^
*SDC2*	34.0	9.7	1.8	1.6	18.5	5.50 × 10^−5^
*IL33*	17.4	10.4	0.4	0.5	46.1	5.04 × 10^−5^
*GFAP*	119.7	113.1	8.5	11.9	14.2	5.91 × 10^−5^
*SOX9*	50.5	18.6	2.2	1.9	22.7	5.52 × 10^−5^
*SPARCL1*	1913.8	576.8	104.0	137.6	18.4	1.47 × 10^−5^
*IL17D*	21.7	4.8	1.9	1.7	11.2	5.52 × 10^−5^
*ID4*	22.5	7.8	0.4	0.4	51.3	5.20 × 10^−5^
*GPC4*	3.5	1.0	0.2	0.2	17.4	4.25 × 10^−5^

The human CNS RNA-Seq database published by the Ben Barres group [49,50] was used to determine the level of astrocyte specificity in functionally-relevant genes that co-expressed with *Cx43*/*GJA1* in spinal cord periplaques. Measures in other CNS cells include pooled results obtained by RNA-Seq on human neurons, endothelial cells, oligodendrocytes and microglia. Statistical comparisons were performed with a Mann–Whitney test. SD: standard deviation; FKPM: fragments per kilobase of exon per million fragments.

**Table 5:** List of interleukins that are constitutively expressed by normal mature human astrocytes.

Gene Symbol	Astrocytes (Mean FPKM)	SD	Other CNS Cells (Mean FPKM)	SD	Fold Change	*p*-Value
*IL17D*	21.7	4.8	1.9	1.7	11.2	5.52 × 10^−5^
*IL33*	17.4	10.4	0.4	0.5	46.1	5.04 × 10^−5^
*IL1B*	7.0	5.9	219.4	344.3	0.0	0.002
*IL6*	2.1	3.1	8.2	9.6	0.3	0.095
*IL9*	0.7	0.7	0.2	0.1	4.3	0.0049
*IL18*	0.7	0.6	14.1	20.1	0.0	0.01
*IL1A*	0.6	0.4	28.9	49.9	0.0	0.01

The human CNS RNA-Seq database published by the Ben Barres group [49,50] was used to determine the constitutive expression and level of astrocyte specificity in 33 human interleukins. Measures in other CNS cells include pooled results obtained by RNA-seq on human neurons, endothelial cells, oligodendrocytes and microglia. Statistical comparisons were performed with a Mann–Whitney test. SD: standard deviation; FKPM: fragments per kilobase of exon per million fragments.

## Supplementary Materials

Supplementary materials can be found at www.mdpi.com/1422-0067/18/10/2097/s1.

## Abbreviations

MS	Multiple sclerosis
RRMS	Relapsing-remitting multiple sclerosis
SPMS	Secondary progressive multiple sclerosis
PPMS	Primary progressive multiple sclerosis
NAWM	Normal-appearing white matter
